# EST-based *in silico* identification and *in vitro* test of antimicrobial peptides in *Brassica napus*

**DOI:** 10.1186/s12864-015-1849-x

**Published:** 2015-09-02

**Authors:** Tao Ke, Huihui Cao, Junyan Huang, Fan Hu, Jin Huang, Caihua Dong, Xiangdong Ma, Jingyin Yu, Han Mao, Xi Wang, Qiuhong Niu, Fengli Hui, Shengyi Liu

**Affiliations:** Key Laboratory of Biology and Genetic Improvement of Oil Crops, Ministry of Agriculture, Oil Crops Research Institute of CAAS, Wuhan, 430062 P. R. China; College of Life Science and Technology, Nanyang Normal University, Nanyang, 473061 China; Hubei Collaborative Innovation Center for Green Transformation of Bio-Resources, Hubei University, Wuhan, 430062 P. R. China

**Keywords:** Expressed sequence tag, Antimicrobial peptides, Antimicrobial activities, *Brassica napus*, *In silico* identification, Highly efficient AMP prokaryotic expression system

## Abstract

**Background:**

*Brassica napus* is the third leading source of vegetable oil in the world after soybean and oil palm. The accumulation of gene sequences, especially expressed sequence tags (ESTs) from plant cDNA libraries, has provided a rich resource for genes discovery including potential antimicrobial peptides (AMPs). In this study, we used ESTs including those generated from *B. napus* cDNA libraries of seeds, pathogen-challenged leaves and deposited in the public databases, as a model, to perform *in silico* identification and consequently *in vitro* confirmation of putative AMP activities through a highly efficient system of recombinant AMP prokaryotic expression.

**Results:**

In total, 35,788 were generated from cDNA libraries of pathogen-challenged leaves and 187,272 ESTs from seeds of *B. napus*, and the 644,998 ESTs of *B. napus* were downloaded from the EST database of PlantGDB. They formed 201,200 unigenes. First, all the known AMPs from the AMP databank (APD2 database) were individually queried against all the unigenes using the BLASTX program. A total of 972 unigenes that matched the 27 known AMP sequences in APD2 database were extracted and annotated using Blast2GO program. Among these unigenes, 237 unigenes from *B. napus* pathogen-challenged leaves had the highest ratio (1.15 %) in this unigene dataset, which is 13 times that of the unigene datasets of *B. napus* seeds (0.09 %) and 2.3 times that of the public EST dataset. About 87 % of each EST library was lipid-transfer protein (LTP) (32 % of total unigenes), defensin, histone, endochitinase, and gibberellin-regulated proteins. The most abundant unigenes in the leaf library were endochitinase and defensin, and LTP and histone in the pub EST library. After masking of the repeat sequence, 606 peptides that were orthologous matched to different AMP families were found. The phylogeny and conserved structural motifs of seven AMPs families were also analysed. To investigate the antimicrobial activities of the predicted peptides, 31 potential AMP genes belonging to different AMP families were selected to test their antimicrobial activities after bioinformatics identification. The AMP genes were all optimized according to *Escherichia coli* codon usage and synthetized through one-step polymerase chain reaction method. The results showed that 28 recombinant AMPs displayed expected antimicrobial activities against *E. coli* and *Micrococcus luteus and Sclerotinia sclerotiorum strains*.

**Conclusion:**

The study not only significantly expanded the number of known/predicted peptides, but also contributed to long-term plant genetic improvement for increased resistance to diverse pathogens of *B.napus*. These results proved that the high-throughput method developed that combined an *in silico* procedure with a recombinant AMP prokaryotic expression system is considerably efficient for identification of new AMPs from genome or EST sequence databases.

**Electronic supplementary material:**

The online version of this article (doi:10.1186/s12864-015-1849-x) contains supplementary material, which is available to authorized users.

## Background

Gene-encoded anti-microbial peptides (AMPs) are widespread in nature, and are essential lines of host defence against pathogens. These peptides are evidently less susceptible to bacterial resistance than traditional antibiotics, and could form the basis for a new class of therapeutic agents [1]. As eukaryotes, plants have innate AMP defense that usually consists of small Cys- or glycine-rich peptides that are effective against a variety of pathogens. The main classes of AMPs are represented by the alpha/beta-defensins, lipid-transfer proteins (LTP), thionins, cyclotides, snakins, and hevein-like proteins according to their amino acid sequence homologies [2]. Interestingly, a series of novel plant AMPs has been discovered as processed forms of large proteins. Plant AMPs provide novel strategies not only in therapeutic use, but can also potentially increase agricultural yields through phytopathogen or pest control [3].

To date, in spite of the increasing number of reported AMPs from plants, developments in gene expression methodologies and computational algorithms lead to new prospective strategies of biomining AMPs in plant systems. Computational and bioinformatics approaches has allowed the application of silico-associated molecular tools aiming to screen and identify novel potential candidates that code for these peptides, starting from substantial amounts of genomics, proteomics, transcriptomics, metabolomics, and other ‘-omics’ data from various cultivated or wild plants [2]. Expressed sequence tag (EST) databases are increasing in number and size, especially regarding cultivated plants [4]. Currently, more than 63 million ESTs are available through the dbEST entry of GenBank (http://www.ncbi.nlm.nih.gov/dbEST/dbEST). As expected, crop species have been more frequently targeted for AMP research and application due to the highly available molecular data [2]. Recent bioinformatics analyses of sequenced plant genomes have revealed a previously unrecognized abundance of genes encoding AMPs [5].

*B. napus* is one of the most important oil crops worldwide, providing a challenge for understanding innate immune systems and resistances to phytopathogens in Brassicaceae plants. The large number of EST sequences of *B. napus* provides a timely opportunity to discover a complete repertoire of AMP sequences. Candidate AMP-coding genes identified from *in silico* approaches require further biological validation. In this study, after bioinformatics analysis of potential AMP genes from three different *B. napus* EST databases, an effective method has been performed for large-scale validation of the biological activities of these candidate AMP genes [6].

## Results

### EST-based search for discovery of novel *B. napus* AMPs

In this study, a general search aiming to identify AMPs was performed on three different *B. napus* EST databases including: 136,202 ESTs generated from immature seeds of two rapeseed lines [7]; 35,788 ESTs generated from an cDNA library which was constructed from mixed mRNAs of *B. napus* leaves inoculated with *S. sclerotiorum* or treated with chemicals benzothiadiazole (BTH), methyl jasmonate (MeJA), or oxalic acid (OA, a toxin and pathogenicity factor produced by *S. sclerotiorum*); 643,944 ESTs downloaded from the EST database of PlantGDB (refreshed until Oct-1-2009). After screening for low-quality DNA and trimming the vector sequences, the three EST datasets were clustered and produced unigenes using CAP3 program respectively (Table 1). To identify novel AMP genes in the *B. napus*, all the known AMPs from the AMP databank (APD2 database, including 1199 known AMPs sequence, http://aps.unmc.edu/AP/main.php) were individually queried first in the three unigene datasets using the BLASTX program [8] (Additional file 1-blast result). A total of 237 unigenes from *B. napus* leaves that were matched to the known AMP genes had the highest ratio (1.15 %) in this unigene dataset, which is 13 times that of the unigene datasets of *B. napus* seeds (0.09 %) and 2.3 times that of the public EST dataset (Table 1).

**Table 1:** Summary of expressed sequence tags (ESTs) from the four origins

species	No. of sequences generated	No. of high-quality sequences	No. of unigenes	Hit to No. of AMPs (%)
*B. napus* from PlantGDB EST	644,998	632,919	145,002	705(0.49 %)
*B. napus* leaf	35,788	35,756	20,587	237(1.15 %)
*B. napus* (High oil content) seed	78,332	69,938	21,712	19(0.09 %)
*B. napus* (Low oil content) seed	57,870	50,656	13,899	11(0.09 %)
Total	816,988	789,269	201,200	

### Analysis of ESTs related to AMP

A total of 972 unigenes that matched the 27 known AMP sequences were extracted and annotated with gene ontology (GO) terms and the BLAST result against the Swissprot database with an E value cut-off that was equal to or less than 10^−5^ using Blast2GO program (Additional file 2-blast2go result). Among these unigenes, roughly 90 % of each EST library was LTP (32.0 %), defensin (15.7 %), histone (15.7 %), endochitinase (13.5 %), and gibberellin-regulated protein (10.5 %) (Additional file 2-blast2go result). The most abundant unigenes in the leaf library were the endochitinase and defensin genes, which were different from LTP and histone in the pub EST library (Table 2).

**Table 2:** Annotation of unigenes hit to AMP database

Genes	No. of unigenes hit to AMP database			
Dataset	Leaf	Pub	Seed	Total
endochitinase	112	19	0	131
defensin-like protein	64	87	2	153
gibberellin-regulated protein	4	89	9	102
histone	32	112	9	153
LTP	13	292	6	311
others	12	106	4	122
Total	237	705	30	972

After deleting the redundant sequences with 100 % sequence identity, 606 peptide sequences were found, matching the different AMP families (Table 3). All the sequences were new, except for one defensin gene that was completely similar to AMP Rs-AFP1 (APD2 database ID: AP00286).

**Table 3:** The number of potential AMP sequence and families

Total	LTP	Defensin	Snakin	Hipposin	Hevein	Thionin	Other
606	237	106	91	79	37	10	46
	39.1 %	17.5 %	15.0 %	13.0 %	6.0 %	1.6 %	7.6 %

### Phylogeny and motif analysis

**Fig. 1:**
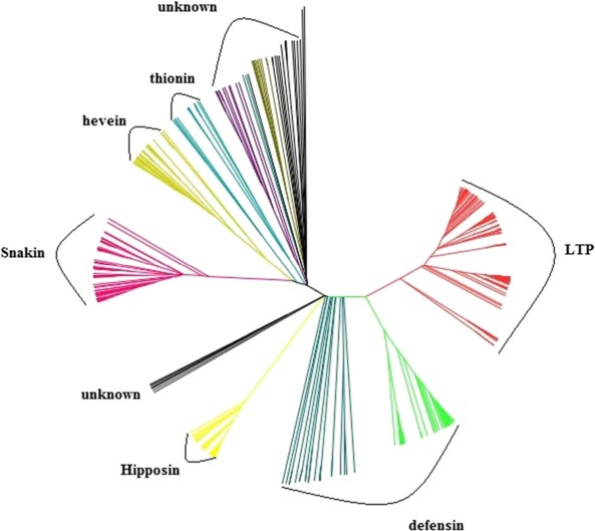
Phylogenetic relationship of all potential AMPs. A multiple sequence alignment of 606 potential AMPs was used to calculate a matrix with the genetic distances for each pair of the sequences. Based on this matrix, successive clustering of lineages was done to construct the unrooted phylogenetic tree of all potential AMPs gene using the maximum-likelihood algorithm with 1000 bootstrap replicates. Only branches supported by a bootstrap value of at least 50 % are indicated. Tree was generated using JalView [9]. The tree includes 606 sequences, and therefore only major nodes and global clusters are depicted. The labeling of the subfamilies is based on the location of AMPs that have experimentally confirmed function

606 potential AMPs were aligned via ClustalW to construct the unrooted phylogenetic tree using the maximum-likelihood algorithm with 1000 bootstrap replicates (Fig. 1). Six groups of known AMP families were found, including the most abundant types (39.1 %) LTP, defensin-like peptides (DLPs), snakin (extracted from gast1 genes), hipposin (histone-derived AMPs, HDAPs), hevein (extracted from endochitinase genes), and thionin. 46 peptides belong to other unknown families are also found in *B. napus*.

**Fig. 2:**
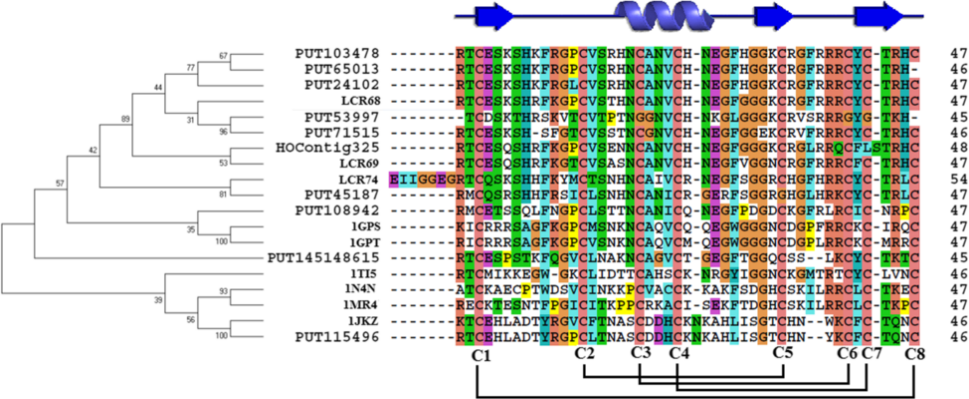
Comparison of amino acid sequences of some defensin-like. Evolutionary relationship is depicted left. Linked bars representing the disulfide bonds arrangement denoted by: C1-C8; C2-C5; C3-C6; C4-C7. Blue helix and arrow respectively represent alpha-helix and beta-strand, which are extracted from the model structure of [PDB:1N4N]. [PDB:1GPS] (γ-1-P thionin: *Triticum aestivum*); [PDB:1GPT] (γ-1-P thionin: *Hordeum vulgare*); [PDB:1JKZ] (Defensin: *Pisum sativum*); [PDB:2GL1] (Defensin: *Vigna radiata*); [PDB:1TI5] (Defensin: *Vigna radiata*); [PDB:1MR4] (Defensin: *Nicotiana alata*); [PDB:1N4N] (Defensin: *Petunia* x *hybrida*). Numbers at the base of each clade correspond to bootstrap means at 1000 replications

Each of the resulting rough set of AMPs family sequence subgroups was separately realigned via ClustalW and visualized using JalView [9]. Defensins are small (~5 kDa) cysteine-rich peptides and consists of a cystine-stabilized αβ fold, in which the buried hydrophobic core is formed by four disulfide bridges that link pairs of the eight conserved cysteines (Cys1 through Cys8). The defensin-like peptides (DLPs) of *B. napus* have four distinct clusters (Additional file 3: Figure S1). All clusters have eight conserved Cys residue motifs forming four disulfide bonds. The evolutionary history of one DLP cluster candidate was reconstructed with MEGA5 [10] at the protein level (Fig. 2). Multiple alignment of this DLP cluster revealed that the consensus pattern C-X5-C-X3-C-X9(10)-C-X6-C-X-C in defensin domain were highly conserved as typically observed in 1TI5 (PDB) from *Vigna radiata*.

LTPs are stabilized by eight conserved Cys residues forming four disulfide bonds, and have a potential glycophosphatidylinositol modification site and a defined number of residues between each two of the eight conserved Cys residues [11–14]. LTPs are categorized into five types or clades by comparison of the LTP genes within and among individual plant species [12]. Type I LTPs and their derivatives are the basic and ubiquitous clades. The major LTPs in *B. napus* belong to LTP type I (roughly 90 %), with a hydrophilic residue such as arginine, glutamate, or lysine at the × position of CXC motif (C6-X-C8 motif) (Additional file 3: Figure S2). Snakin amino acid sequence alignments (Additional file 3: Figure S3) indicate that in addition to the 12 characterized Cys motif, the arginine, valine, and proline residues are highly conserved throughout the family [15, 16]. *B. napus* hevein-like peptide sequences are about 30 amino acids to 45 amino acids long and possess eight unique Cys in a chitin-binding peptide with several strictly conserved residues, serine, and two glycines (Additional file 3: Figure S4) [17, 18]. *B. napus* thionin has six conserved Cys residues, as well as conserved proline and glycine residues (Additional file 3: Figure S6) [19, 20]. A variable number of Cys residues that helped stabilize conserved scaffolds through disulfide bond formation were found [21]. *B. napus* Hipposin (Histone H2A-derived AMP) sequence had a proline hinge without Cys residues (Additional file 3: Figure S5) [22–24]. In addition, a type of proline-and glycine-rich peptide was found, which was similar to animal AMPs and has not been previously reported (Additional file 3: Figure S7).

### Production of fusion proteins with AMPs via a fusion partner EDDIE

**Fig. 3:**
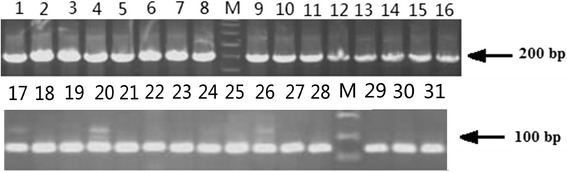
PCR production of AMP genes prepared for expression vector construction. Lane M: molecular mass makers; Lane 1–31: PCR production of AMP genes from Bn1 to Bn31

To investigate the antimicrobial activities of the predicted peptides, 31 potential AMP genes that belong to different AMP families were selected to test their antimicrobial activities after bioinformatics identification (Additional file 4-sequence). The AMP genes were optimized according to *E. coli* codon usage and synthetized through one-step polymerase chain reaction (PCR) technique (Fig. 3). To recover their original activities without additional amino acid residues, each PCR production of AMPs genes was cloned into a unique vector with EDDIE as a fusion partner via an *in vivo* recombination strategy [6].

**Fig. 4:**
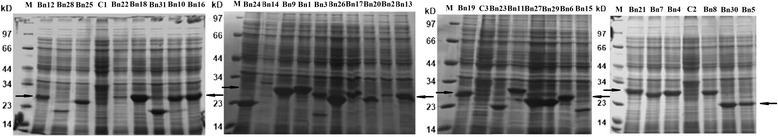
SDS–PAGE analysis of recombinant His-EDDIE-AMPs expressed in *E. coli* BL21 (DE3). Lane M: the molecular weight markers; Lane 1: crude cells extracts of uninduced *E. coli* BL21 containing pET-His-EDDIE-AMP; Lane 2–18: crude cells extracts after 5 h past the induction with IPTG of *E. coli* BL21 containing pET-His-EDDIE-AMP. Arrows indicate the bands of fusion proteins of about 20 kDa

The pET30a/His-EDDIE-AMP plasmids were transformed into the expressing host and cultured under optimized conditions. After the induction by isopropyl β-D-1-thiogalactopyranoside (IPTG), the expression of His-EDDIE-AMP proteins was analyzed using SDS–PAGE (Fig. 4). Fusion proteins of about 20 kDa represented the majority of the insoluble components in the cell lysates. The recombinant His-EDDIE-AMPs were produced as inclusion bodies because of the properties of the fusion partner.

### Generation of AMPs and their activity test

**Fig. 5:**
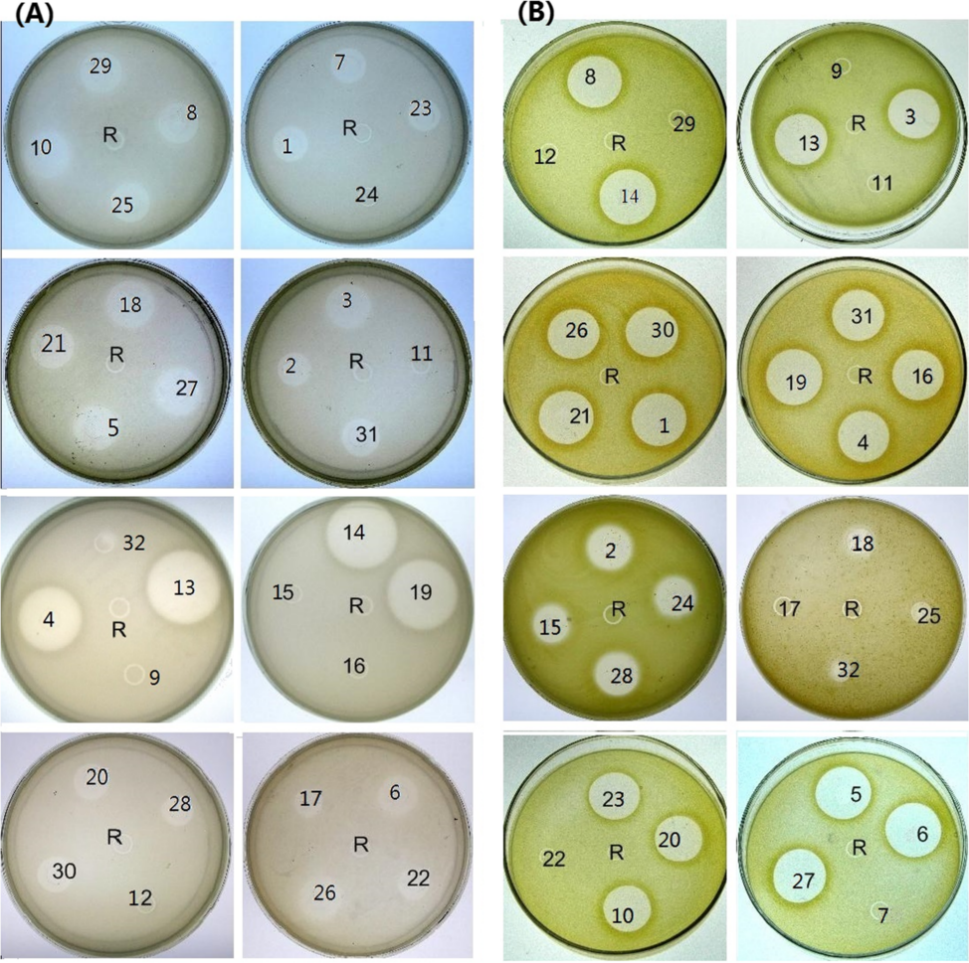
Detection of the antibacterial activities of candidate antimicrobial peptides against *E. coli* and *M. luteus*. R: refolding buffer; **a** antimicrobial activities assay against *E. coli*; **b** antimicrobial activities assay against *M. luteus*; the number in plate from 1 to 31 indicated the name of AMPs from Bn1 to Bn31. The letter “R” in the center of plate indicated the refolding buffer as the control

Purified His-EDDIE-AMP inclusion bodies were diluted in optimized refolding buffer. After *in vitro* refolding, the fusion partner was released from the C-terminal end of the autoprotease through self-cleavage, leaving the AMPs with an authentic N terminus. To examine the antimicrobial activities of the recombinant AMPs, the purified supernatants were analysed using radial diffusion assay. In Fig. 5, a total of 28 recombinant AMPs were clearly bioactive and significantly effective in destroying the sensitive strains (Additional file 5-activities test), but no inhibition zones were seen around the negative control spots. Among these AMPs, three AMPs have only antimicrobial activities to Gram negative strains and other three AMPs have only antimicrobial activities to Gram positive strains. Bn19 have the highest activities to Gram positive and negative strains among these 28 AMPs.

**Fig. 6:**
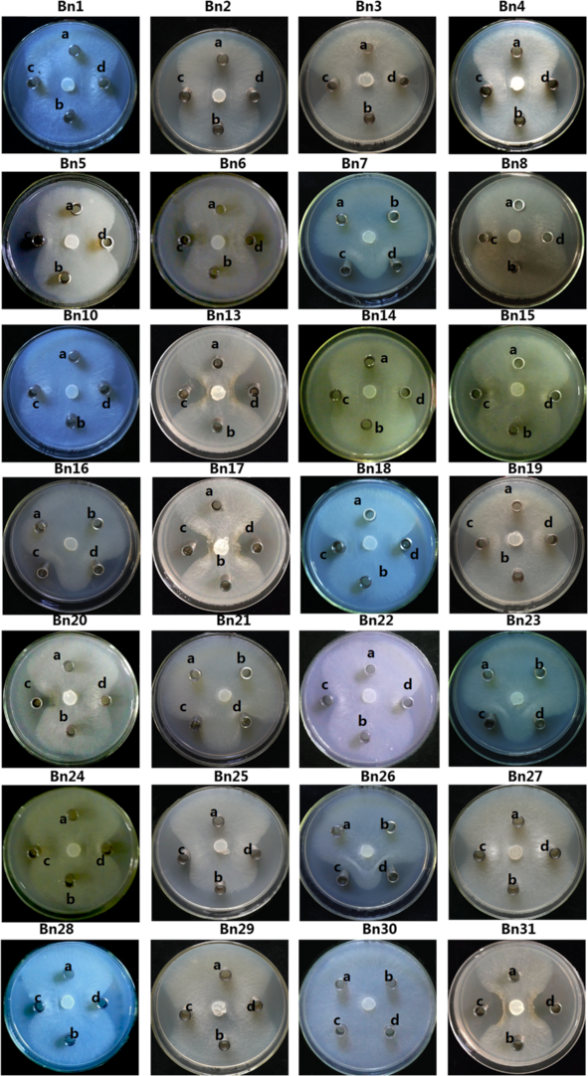
Detection of the antifungal activities of candidate antimicrobial peptides against *S. sclerotiorum*. In the plate containing PDA medium, a mycelial plug was placed in the center. The name of AMPs are indicated upon every plate from Bn1 to PDA medium, a mycelial plug was placed in the center. The name of AMPs are indicated upon every plate from Bn1 to Bn31. The letter “**a**, **b**” in the plates indicated wells with the refolding buffer as the control, “**c**, **d**” indicated wells with antimicrobial peptide samples

The antifungal activity assay for 28 recombinant purified AMPs with antibacterial activities to Gram positive or negative strains was carried out with *S. sclerotiorum* strains, a main agronomically phytopathogen of *B. napus*. As illustrated in Fig. 6, the growth of the tested filamentous fungal strain was inhibited by 27 AMPs, but it can grow normally with Bn30 and the control refolding buffer.

A total of 28 AMPs, including nine heveins, five defensins, three hipposins, three thionins, three snakins, and four LTPs, showed activities against sensitive strains. A new AMP sequence Bn19 with rich proline residues similar to SP-B (pigs, animals, and AP00889) [25] was classified as a new member of the proline-rich antimicrobial peptide family.

## Discussion

By using integrated computational approaches to systemically mine the *B. napus* EST sequence, the first *B. napus* AMP repertoire was established. The members exhibit extensive sequence and structural diversity, and can be distinguished into multiple molecular types. The 606 *B. napus* non-redundant AMPs were organized in seven subfamily types, namely, DLPs with Cys-stabilized alpha-helical and beta-sheet (CSab) fold, LTP, thionin, Hipposin, hevein peptide, snakin, and proline- or glycine-rich peptides [26]. Results of the bioinformatics and phylogenetic analyses of the primary structures of the *B. napus* candidates support their role as antimicrobials. Furthermore, the Cys motif characteristics and other conserved residues of the main AMP families, such as defensin, thionin, and LTP gene families, were summarized.

AMP candidate genes are more substantially abundant in *B. napus* leaves in response to the pathogene (*S. sclerotiorum*) and signaling compounds compared with *B. napus* seeds and public EST dataset. The most abundant unigenes in the leaf library are endochitinase-derived hevein and defensin genes, indicating that these genes are necessary in the protection against potential pathogen, defensins and other AMPs often expressed in an organ- or tissue-specific manner.

Most of the candidate peptide sequences are new AMP genes that have no proven antimicrobial activities. Flowering plants have been previously shown to possess large gene families encoding LTPs [14]. The results in this research also show that LTP is the most abundance type in B. napus. Next to their inducibility upon pathogen infection, LTP genes are also responsive to abiotic stresses like drought, cold and salt [27], and perhaps necessary for pollen adherence to the stigma during pollen elongation in some flowering plants [28]. A defensin from cowpea seeds was assessed on its putative alpha-amylase inhibitory action probably involved in protection against pests [29].

In this study, an efficient method was used for cloning and expressing AMP genes. This expression system is more efficient and is very useful for constructing genome-scale clone resources that facilitate AMP functional analysis. This approach, coupled with bioinformatics analyses of the genome and EST sequence data, will be useful in screening for new AMPs. Such tools may contribute in overcoming problems associated with yield, storage, and processing, thereby improving crop resistance and providing novel strategies not only in medicine but can potentially increase agricultural yields by phytopathogen or pest control.

Thirty-one potential AMPs in this study were detected and 28 of them were confirmed to be novel AMPs of *B. napus* using antibacterial activity assay. This finding indicates that large AMP genes are still undiscovered. 27 AMP candidate genes were also approved with strong activities to control the main fungal pathogens of *B. napus*, *S. sclerotiorum*. These AMPs will be further proved in enhanced crop resistance to pathogen attack through genetic breeding and transgenic manipulation in the future.

## Conclusions

This study provides new insights and fundamental information on *B. napus* AMP gene families. Based on their existence in natural host defence systems and their different modes of actions relative to commercial antibiotics, antimicrobial peptides represent new hope in discovering novel antibiotics against multi-resistant bacteria.

## Methods

### Materials

*E. coli* XL-GOLD (Stratagene, USA) was used as the host for subcloning and plasmid amplification. *E. coli* BL21 (DE3) was used as the host for expressing the recombinant protein. *E. coli* ATCC2592 and *M. luteus* ACCC11001, *S. sclerotiorum* were used as indicators in the antimicrobial assay for the antimicrobial peptides. pET30a (Novagen, Madison, WI, USA) was used as a vector construction and recombinant protein expression plasmid. The restriction enzymes *Nde*I and *Sal*I were purchased from Takara (Dalian, China).

### Database searches and peptide prediction

The *B. napus* ESTs used for the prediction of AMP genes were collected from three different EST data sets, namely, data for assembled unigenes (the PUT, PlantGDB-generated Unique Transcript) downloaded from the PlantGDB (refreshed until Oct-1-2009), *B. napus* seed ESTs from *B. napus* cv. ZY036 (high-oil contents, HO) and *B. napus* cv. 51070 (low-oil contents, LO), by 454 sequencing (2 weeks after flowering) [7], and *B. napus* leaf ESTs generated from mixed mRNAs of *B. napus* cv. M083 and *B. napus* cv. Zhongshuang9 leaves inoculated with *S. sclerotiorum* or treated with chemicals benzothiadiazole (BTH), methyl jasmonate (MeJA), or oxalic acid (OA, a toxin and pathogenicity factor produced by *S. sclerotiorum*).

The strategies for gene discovery used in this study are provided in the supplementary information (see Additional file 6). Database searches were conducted using methods modified from several recent publications [30–36]. The programs BLASTP and BLASTX were used to mine ESTs encoding putative *B. napus* peptide precursors via queries using known AMP sequences. All candidate nucleotide sequences were translated to amino acids using getORF [37]. All hits were fully translated and manually checked for homology with the target query.

### Evolutionary analysis

The retrieved sequences were aligned to one another with ClustalW (Version 1.82) [38], and gapped positions were omitted from subsequent analyses [39]. A multiple sequence alignment of 606 potential AMPs was used to calculate a matrix with the genetic distances for each pair of the sequences. Based on this matrix, successive clustering of lineages was done to construct the unrooted phylogenetic tree of all potential AMPs gene using the maximum-likelihood algorithm with 1000 bootstrap replicates. Only branches supported by a bootstrap value of at least 50 % are indicated. Tree was generated using JalView [9]. Each of the resulting rough set of AMPs family sequence subgroups was separately realigned via ClustalW and visualized using JalView [9].

### Construction of the AMP expression vector with EDDIE as a fusion partner

A total of 31 new potential AMPs were selected from different AMP families to determine their activities against bacteria. The AMP sequences were optimized according to *E. coli* codon usage (Additional file 4-sequence). Two, four, or six primers were used to synthesize each AMP gene in a one-step PCR (Additional file 7-primers). The PCR reaction was performed for 25 cycles, each cycle consisting of 30 s at 94 °C, 30 s at 62 °C, and 7 min at 72 °C. Each synthetic gene was then cloned into the pET30a/His-EDDIE-GFP vector via an *in vivo* recombination strategy [6]. Each AMP was then expressed as an Npro fusion protein in *E. coli*. White colonies were selected and then sequenced to ensure that the coding sequence was appropriate. The resulting plasmids were named pET30a/His-EDDIE-AMPs, respectively.

### Expression and purification of fusion protein

The pET30a/His-EDDIE-AMP plasmids were transformed into the expression host *E. coli* BL21 (DE3) (Novagen, Madison, WI, USA). A colony was used to inoculate 50 mL of LB (1 % Bacto-tryptone, 0.5 % yeast extract, and 8 mM NaCl) medium supplemented with 50 μg/mL kanamycin, and grown overnight at 37 °C in a shaking incubator. The fully grown culture was mixed with 1 L LB medium with the same antibiotics the following morning. The culture was grown at 25 °C, and IPTG was added to a final concentration of 1 mM when the OD_600_ reached 0.5. The culture was harvested 5 h later, and the cells were washed and resuspended in 30 mL phosphate-buffered saline (NaCl 137 mM, KCl 2.7 mM, Na_2_HPO_4_ 4.3 mM, and KH_2_PO_4_ 1.4 mM; pH7.2–7.4). The cells were lysed through freeze-thawing, and DNA was fragmented through ultrasonication. The insoluble inclusion bodies were isolated by centrifugation at 14,000 x g at 4 °C for 30 min. The pellets were washed three times with washing buffer (10 mM Tris/HCl, pH7.6; 200 mM NaCl, 2 mM 2-mercaptoethanol, and 1 % Triton X-100) and then solubilized in the denaturation buffer (8 M urea; 20 mM Tris–HCl, pH7.6; and 5 mM 2-mercaptoethanol).

### Antimicrobial assays

Purified His-EDDIE-AMPs inclusion bodies were refolded by rapid 1:50 dilution in an optimized refolding buffer (500 mM NaCl, 20 mM Tris, 2 mM EDTA, 5 % glycerol, 10 mM DTT, 0.01 % Tween-20, pH 7.5) and incubated at an appropriate temperature without stirring. During refolding, EDDIE restored its correct conformation and self-cleaved at the specific sites, releasing AMPs from the fusion bodies. Renatured protein solution was then clarified by centrifugation at 15,000 x g at 4 °C for 30 min. The insoluble sample was removed by filtering through a 0.45 μm membrane, leaving the AMPs in the supernatant. The supernatants were transferred to a Ni-NTA His-bind column for purification.

Standard SDS–PAGE (12 % gel) was used to assay the fusion proteins. The band density was analyzed using a GEL-DOC 2000 gel documentation system (BIO-Rad, Hemel Hempstead, UK). Quantity One software version 4.4.0 was used to determine the fraction of the target protein. EDDIE protein was quantified using a bicinchoninic acid protein assay kit (Pierce, Rockford, IL, USA).

The antimicrobial activities of recombinant AMPs were detected using a radial diffusion assay [40]. *E. coli* ATCC2592 and *M. luteus* ACCC11001 were grown on the mid-logarithmic phase and washed. Approximately 2 × 10^6^ cfu/mL bacteria were incorporated into a thin (1.2 mm) agarose underlay gel containing 1 % (*w*/*v*) agarose. Holes with 3.5 mm diameter were punched into the solidified agarose and were filled with 100 μL of AMP sample. After the plates were incubated at 37 °C for 12 h, the diameter of the clear zone surrounding each well was measured to evaluate the antimicrobial activity. The refolding buffer was used as negative controls. The above assays were performed in triplicate.

The antifungal activity of the purified products was assayed using an ultra-sensitive radial diffusion method on thin potato plates (200 g potato, 20 g glucose, 15–20 g agar powder, 1 L double-distilled water) seeded with filamentous fungi. Briefly, 9-cm plates were poured with an underlay potato medium, and *S. sclerotiorum* on a 1-cm diameter potato medium were seeded on the center of the plates, which were then incubated at 22 °C until hypha grew to 2 cm in diameter. Two hundred microliters of the test sample and negative control (refolding buffer) was placed beside the filamentous fungi, and the plates were incubated at 22 °C for 72 h; the size of the clear area around the filamentous fungi was then measured.

## Additional files

Additional file 1:
**Blast result of four** ***B. napus*** **ESTs dataset against to known AMPs.** (XLSX 85 kb) Additional file 2:
**The annotation with Gene ontology (GO) terms results of unigenes which matches to AMPs and the blast result against to the swissprot database with an E value cut-off equal or less than 10** ^**−5**^ **using Blast2GO program.** (XLSX 110 kb) Additional file 3:
**Figure S1-S7.** Multiple sequence alignments of different AMPs families. Each of the resulting rough set of AMPs family sequence subgroups was separately realigned via Clustal W and via Jalview. **S1:** defesin family; **S2:** LTP family; **S3:** snakin family; **S4:** hevein family; **S5:** hipposin family; **S6:** thionin family; **S7:** unknown family. (ZIP 3830 kb) Additional file 4:
**Thirty-one Sequences of the AMP candidate for activities conform.** (XLSX 13 kb) Additional file 5:
**Thirty-one recombinant AMP activities test for Gram + and Gram- strain halo diameter.** (XLSX 9 kb) Additional file 6:
**Strategy of database searches of putative** ***B. napus*** **antimicrobial peptides.** (DOC 43 kb) Additional file 7:
**The PCR primers used in this work.** (XLSX 13 kb)
