# Supplementary figures and images for: EST-based in silico identification and in vitro test of antimicrobial peptides in Brassica napus
Source: BMC Genomics. 2015 Sep 2;16(1):653. doi: 10.1186/s12864-015-1849-x (PMC4557752; doi:10.1186/s12864-015-1849-x)

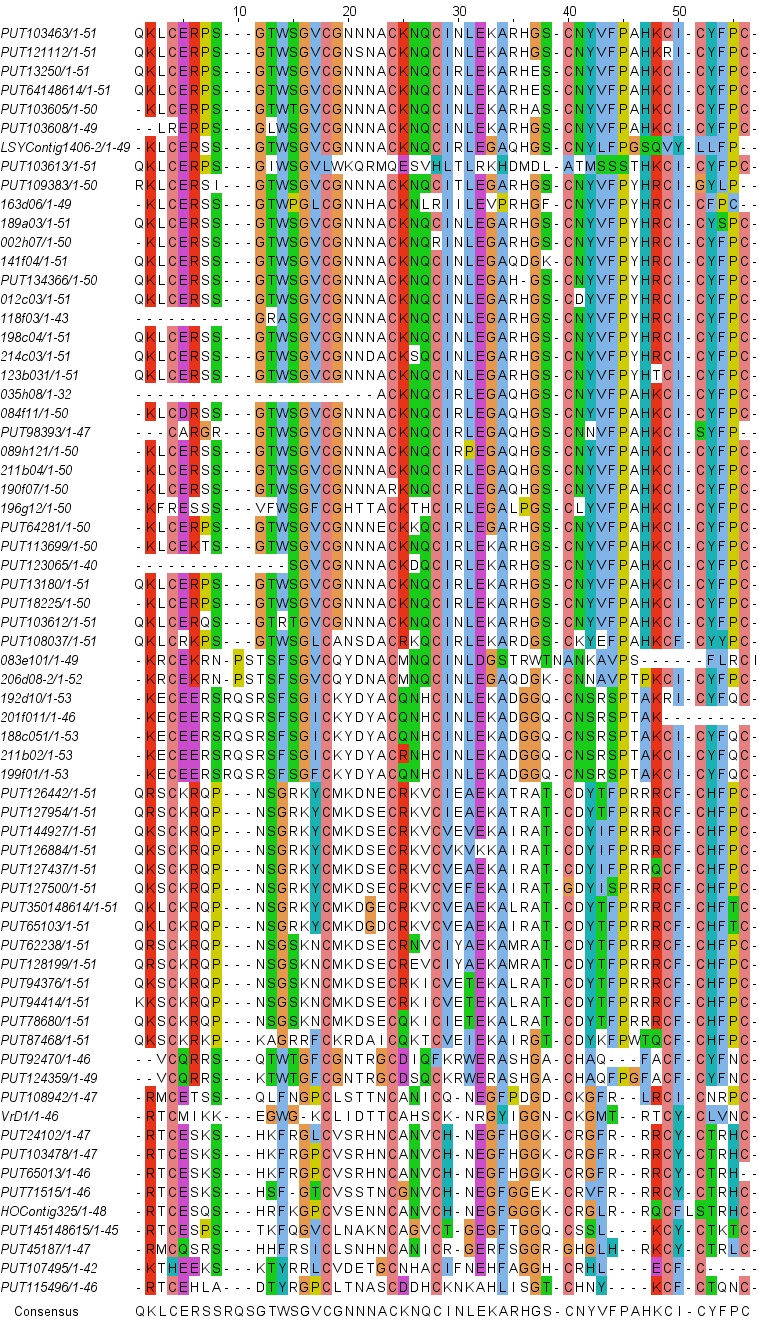

Supplement: Additional file 3: — Figure S1-S7. Multiple sequence alignments of different AMPs families. Each of the resulting rough set of AMPs family sequence subgroups was separately realigned via Clustal W and via Jalview. S1: defesin family; S2: LTP family; S3: snakin family; S4: hevein family; S5: hipposin family; S6: thionin family; S7: unknown family. (ZIP 3830 kb) [file 12864_2015_1849_MOESM3_ESM.zip › 1393246658137933_add7/1393246658137933_figS1.jpeg]

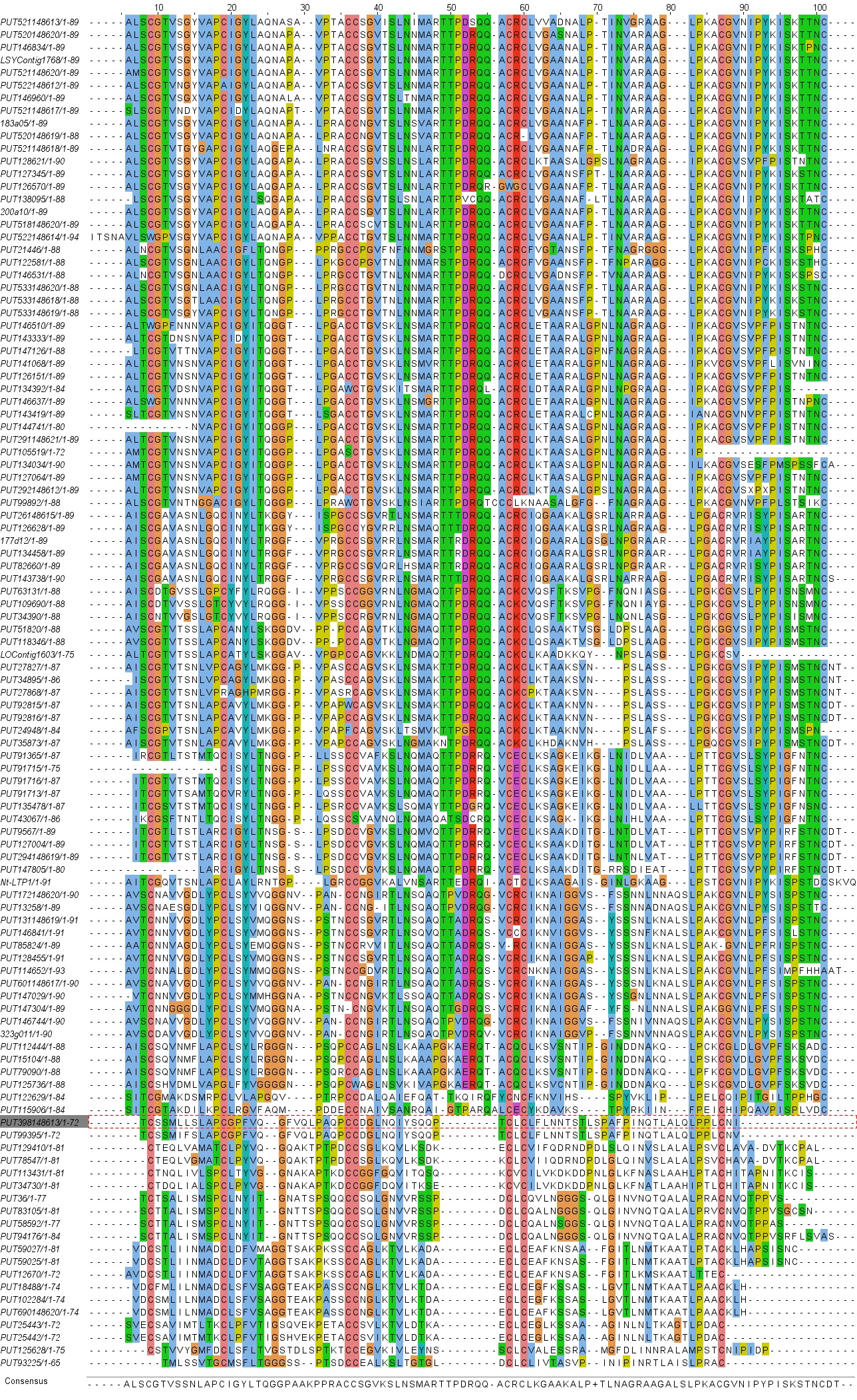

Supplement: Additional file 3: — Figure S1-S7. Multiple sequence alignments of different AMPs families. Each of the resulting rough set of AMPs family sequence subgroups was separately realigned via Clustal W and via Jalview. S1: defesin family; S2: LTP family; S3: snakin family; S4: hevein family; S5: hipposin family; S6: thionin family; S7: unknown family. (ZIP 3830 kb) [file 12864_2015_1849_MOESM3_ESM.zip › 1393246658137933_add7/1393246658137933_figS2.jpeg]

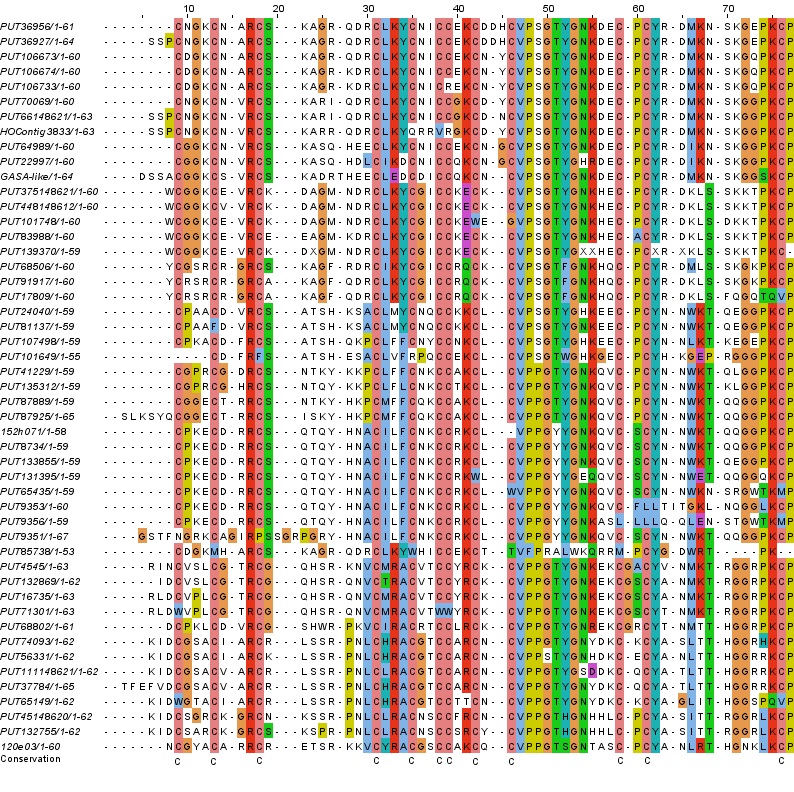

Supplement: Additional file 3: — Figure S1-S7. Multiple sequence alignments of different AMPs families. Each of the resulting rough set of AMPs family sequence subgroups was separately realigned via Clustal W and via Jalview. S1: defesin family; S2: LTP family; S3: snakin family; S4: hevein family; S5: hipposin family; S6: thionin family; S7: unknown family. (ZIP 3830 kb) [file 12864_2015_1849_MOESM3_ESM.zip › 1393246658137933_add7/1393246658137933_figS3.jpeg]

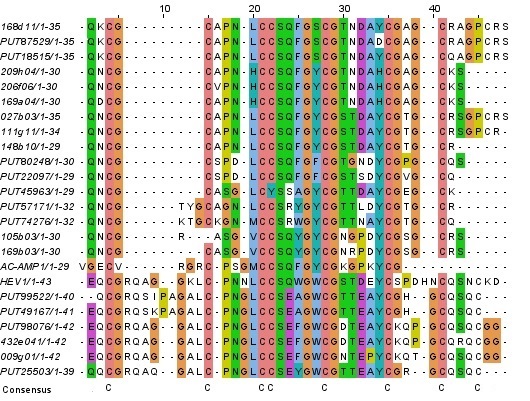

Supplement: Additional file 3: — Figure S1-S7. Multiple sequence alignments of different AMPs families. Each of the resulting rough set of AMPs family sequence subgroups was separately realigned via Clustal W and via Jalview. S1: defesin family; S2: LTP family; S3: snakin family; S4: hevein family; S5: hipposin family; S6: thionin family; S7: unknown family. (ZIP 3830 kb) [file 12864_2015_1849_MOESM3_ESM.zip › 1393246658137933_add7/1393246658137933_figS4.jpeg]

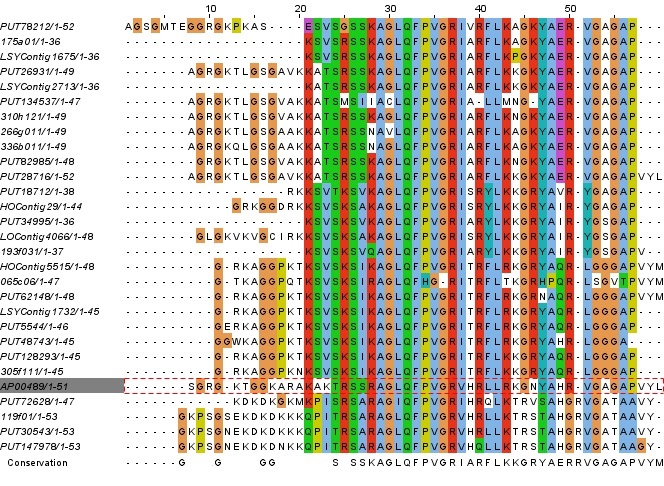

Supplement: Additional file 3: — Figure S1-S7. Multiple sequence alignments of different AMPs families. Each of the resulting rough set of AMPs family sequence subgroups was separately realigned via Clustal W and via Jalview. S1: defesin family; S2: LTP family; S3: snakin family; S4: hevein family; S5: hipposin family; S6: thionin family; S7: unknown family. (ZIP 3830 kb) [file 12864_2015_1849_MOESM3_ESM.zip › 1393246658137933_add7/1393246658137933_figS5.jpeg]

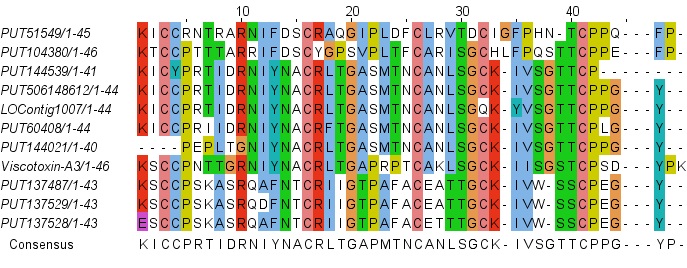

Supplement: Additional file 3: — Figure S1-S7. Multiple sequence alignments of different AMPs families. Each of the resulting rough set of AMPs family sequence subgroups was separately realigned via Clustal W and via Jalview. S1: defesin family; S2: LTP family; S3: snakin family; S4: hevein family; S5: hipposin family; S6: thionin family; S7: unknown family. (ZIP 3830 kb) [file 12864_2015_1849_MOESM3_ESM.zip › 1393246658137933_add7/1393246658137933_figS6.jpeg]

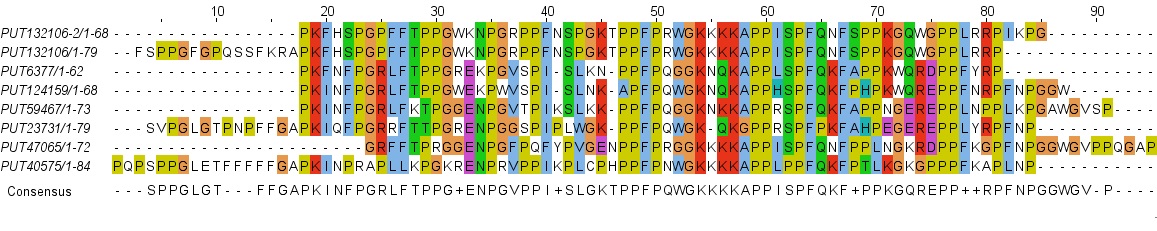

Supplement: Additional file 3: — Figure S1-S7. Multiple sequence alignments of different AMPs families. Each of the resulting rough set of AMPs family sequence subgroups was separately realigned via Clustal W and via Jalview. S1: defesin family; S2: LTP family; S3: snakin family; S4: hevein family; S5: hipposin family; S6: thionin family; S7: unknown family. (ZIP 3830 kb) [file 12864_2015_1849_MOESM3_ESM.zip › 1393246658137933_add7/1393246658137933_figS7.jpeg]
