# Supplementary material for: EST-based in silico identification and in vitro test of antimicrobial peptides in Brassica napus
Source: BMC Genomics. 2015 Sep 2;16(1):653. doi: 10.1186/s12864-015-1849-x (PMC4557752; doi:10.1186/s12864-015-1849-x)
Supplement: Additional file 6: — Strategy of database searches of putative B. napus antimicrobial peptides. (DOC 43 kb) [file 12864_2015_1849_MOESM6_ESM.doc]

**Additional file 5** Strategy of database searches of putative *Brassica napus* antimicrobial peptides.

**known antimicrobial peptides from AMP Database**

***BLASTP***

***BLASTX***

***Brassica napus* EST database**

**(from NCBI GenBank and sequence of**

**our seeds and leaf cDNA library)**

**Putative *Brassica napus* antimicrobial peptides precursors**

***Functional Annotation & getORF***

**Putative *Brassica napus* antimicrobial peptides amino acid sequences**

***Multiple alignment***

***Phylogenetic construction***

**new *Brassica napus* antimicrobial peptides**

***characteristics of new antimicrobial***

***peptides candidiate***

## *Construction of the AMP expression vector and the Activities confirm*
